# Evaluating Diastolic Dysfunction as an Indicator of Cirrhotic Cardiomyopathy in Decompensated Chronic Liver Disease

**DOI:** 10.7759/cureus.63388

**Published:** 2024-06-28

**Authors:** Prashant Kumar, Amish Shikoh, Neha Rani

**Affiliations:** 1 Department of Cardiology, Rajendra Institute of Medical Sciences, Ranchi, IND; 2 Department of Cardiology, North Eastern Indira Gandhi Regional Institute of Health and Medical Sciences, Shillong, IND; 3 Department of Dermatology, Medini Rai Medical College and Hospital, Medininagar, IND

**Keywords:** child turcotte pugh score, diastolic dysfunction (dd), left ventricular diastolic dysfunction (lvdd), decompensated chronic liver disease (dcld), cirrhotic cardiomyopathy (ccm), cirrhosis

## Abstract

Background: The clinical syndrome of cirrhotic cardiomyopathy (CCM) occurs quite frequently in decompensated chronic liver disease (DCLD) patients without any prior incidence. The compromised life expectancy under such conditions was the key that prompted us to conduct this study.

Purpose: This study was planned to study the prevalence of diastolic dysfunction in chronic liver disease patients, to understand the diagnostic criteria of left ventricular diastolic dysfunction (LVDD) in cirrhotic patients, and to evaluate its occurrence as an early indicator of CCM.

Methods: A hospital-based, cross-sectional study was conducted on 158 patients, admitted to the Department of Medicine, Rajendra Institute of Medical Sciences, Ranchi, India, who conformed to our criteria for inclusion and exclusion. The study period was for 18 months. The subjects were clinically and radiologically diagnosed with chronic liver disease. Regression analysis for variables was performed to score the effects of potential variables with outcomes for diastolic dysfunction (DD) prediction.

Results: Out of 158 patients, 116 belonged to the age group of 31-60 years, pronouncing age to be a significant factor for LVDD. Fifty-three subjects had serum bilirubin levels >2mg/dL and we found serum bilirubin levels to bear a significant correlation with LVDD by exhibiting a p-value <0.0001. Both the Child-Turcotte-Pugh score class (p-value=0.0180) and QTc (p-value <0.0001) bear significant correlation with the development of LVDD, which is also evident from their area under the curve (AUC) values of 0.64 in the receiver operating characteristic (ROC) curve.

Conclusion: Our study concludes that LVDD is an early indicator for assessing the severity of liver cirrhosis in DCLD. The correlation of DCLD with prolonged QTc could predispose patients with DCLD to ventricular arrhythmias. Hence, such patients should undergo serum bilirubin tests, and electrocardiographic checks at regular intervals for early detection, to increase their overall survival rates.

## Introduction

Cirrhosis, a term initiated by French physician, René Laënnec in 1819, is typified by histological changes in the hepatocytic regenerative islands, showing nodules and widespread fibrous septae [1]. It is one of the leading causes of global health losses which is preventable but is underappreciated. Cirrhosis accounts for 2.4% of global deaths [2] which may rise in the future as per WHO estimates. One of the prognoses of cirrhosis or decompensated liver disease is cirrhotic cardiomyopathy (CCM). It is a complex myocardial disorder of the heart that limits the ability of the heart to pump well and frequently leads to arrhythmia. The American Heart Association outlines cardiomyopathy as a diverse range of myocardial abnormalities associated usually with either mechanical and/or electrical anomaly, exhibiting inappropriate dilatation or ventricular hypertrophy, generally due to many causes [3]. The major pathophysiologic abnormalities observed are cardiac electrophysiological anomalies, structural as well as functional ventricular anomalies, and responses linked with pharmacological, physiological, or surgical stress. Cardiomyopathies frequently progress to heart failure-related disabilities and cardiovascular death.

CCM arises in cirrhotics without any prior etiology and estimates show the presence of this condition in almost half of cirrhosis patients. CCM significantly reduces life expectancy. The Cirrhotic Cardiomyopathy Consortium in 2019 and the World Congress of Gastroenterology in 2005 enlisted echocardiographic evaluation, especially the diastolic function as one of the criteria of CCM [4]. Cardiac contractility is compromised due to systolic and diastolic dysfunction (DD) resulting from electrophysiological malfunctioning. The American Society of Echocardiography defines left ventricular diastolic dysfunction (LVDD) by the presence of mitral inflow patterns, left atrial (LA) volume index ≥34 mL/m^2^, septal e’ 8cm/sec, and lateral e’ <10cm/sec. Hence, tissue Doppler imaging studies can more appropriately detect LVDD. Conditions of hemodynamic stress like exercise, specific medications, [5] liver transplantation (LTx), and transjugular intrahepatic portosystemic shunt (TIPS) insertion complicate heart function [6,7]. Portal hypertension, one of the outcomes of cirrhosis, increases total blood volume and splanchnic blood flow but reduces circulating blood volume. This leads to total peripheral resistance, higher cardiac output, and decreased arterial pressure. Despite the increased baseline cardiac output, these patients exhibit attenuated systolic and diastolic function due to physiological, pharmacological, and surgical stresses, and also cardio-electrical anomalies like QTc prolongation. The impending disorder is exacerbated by the attack of pro-inflammatory cytokines like interleukin (IL)-6, IL-1β, and tumour necrosis factor-α (TNFα), and vasoactive peptides that damage the cardiac myocytes. Aberration in collagen composition, titin phosphorylation, and cardiomyocyte sarcolemma membrane fluidity are the mechanistic pathways of LVDD [4] which has also been demonstrated in rat cirrhotic models [6].

The structural and functional cardiac activities change over time with the progression of cirrhosis and its outcome. Hence, we have aimed to understand the prevalence of DD in cirrhotic patients based on demographical, etiological, clinical, and biochemical parameters and its occurrence as an early indicator of CCM.

## Materials and methods

Study design

This hospital-based, cross-sectional study was undertaken in the Department of Medicine at Rajendra Institute of Medical Sciences (RIMS), Ranchi, India. A sample size of a total of 158 liver cirrhosis patients, who were willing to take part in the study, were involved. Subjects were clinically and radiologically diagnosed with liver cirrhosis and admitted to the Department of Medicine.

The study was conducted on liver cirrhosis patients who fulfilled all the exclusion and inclusion criteria. The objective was to analyze the prevalence of CCM in decompensated chronic liver disease (DCLD) and to find a correlation and a pattern of distribution between demographical, etiological, clinical, and biochemical parameters, and LVDD. Additionally, we wanted to establish a correlation between the severity of the liver disease by the Child-Turcotte-Pugh (CTP) scoring system and LVDD and hence establish DD as an early indicator of CCM. All 158 patients were evaluated for QTc prolongation by ECG and LVDD by conventional echocardiography for the CCM screening.

The current work was taken up after obtaining approval from the Institutional Ethical Committee, RIMS, Ranchi, Jharkhand, India (Memo No.: 77 IEC.RIMS, Dated: 20.06.22). The patients and their relatives were counselled; informed, written, and signed consent was taken from them. Every participant had the choice to pull out of the study at any time without citing any reason or without prior notice. The study was conducted for a period of 18 months on the patients who met both the inclusion and the exclusion criteria. The inclusion criteria comprised patients with liver cirrhosis but above the age of 18 years admitted to RIMS, irrespective of their gender. This population also included patients who were dependent on alcohol. Exclusion criteria consisted of patients not willing to take part in this study, patients not able to give consent, patients with hypertension, cardiovascular ailments like congenital heart disease, coronary artery disease, rheumatic heart disease, patients with diabetes mellitus, hypo/hyperthyroidism/other endocrine disorders, with severe anaemia and patients with any terminal illness. Sample collection was done only after obtaining ethical clearance. A total of 10 mL of blood was drawn for haematological tests.

2D echocardiography and 12-lead ECG tests were done to evaluate cardiac dysfunctions. As per the American Society of Echocardiography guidelines, all measurements were recorded by one operator. Patients were classified into two groups, with and without LVDD, as per the guidelines, for evaluation of the left ventricular diastolic function by echocardiography. The patients in the latter group, without LVDD, were asymptomatic for Grade 1 DD. Patient data were gathered and recorded on prepared proforma for every patient. Clinical history was recorded after a thorough clinical assessment.

Statistical analyses

All the data were noted and tabulated on an MS Excel sheet (Microsoft® Corp., Redmond, USA) and subjected to descriptive statistical analysis. Chi-square test and multivariable logistic regression analyses were performed with data to predict the outcome of a response variable. The multivariable model was evaluated by the logistic receiver operating curve (ROC). The Statistical Package for the Social Sciences (IBM SPSS Statistics for Windows, IBM Corp., Version 21.0, Armonk, NY) was used for all statistical analyses.

## Results

The study population included 158 patients who were diagnosed with chronic liver disease based on clinical, biochemical, and radiological evidence. All 158 patients were assessed for QTc prolongation by ECG and LVDD by conventional echocardiography for CCM screening.

Age distribution

**Figure 1:**
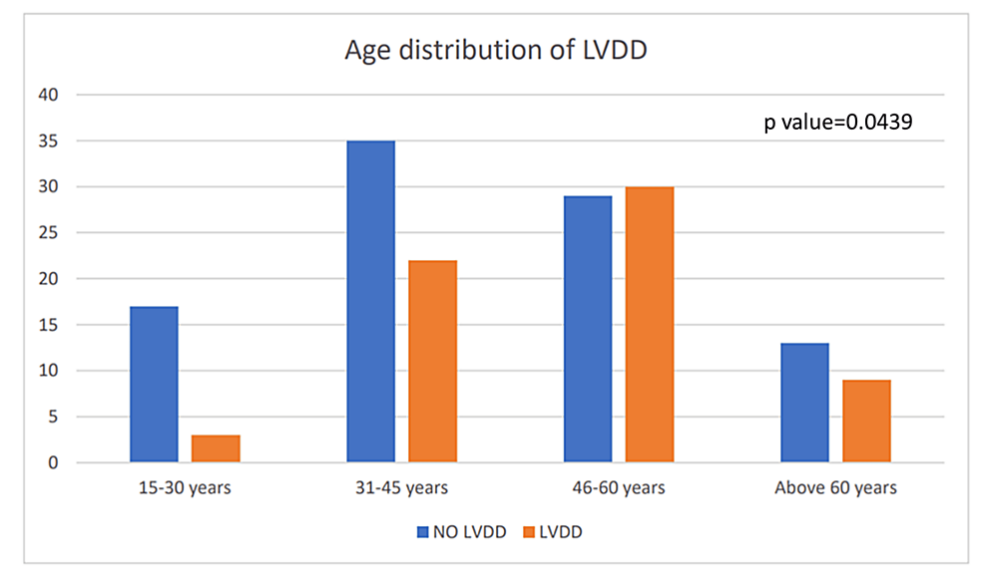
Graph showing the distribution of left ventricular diastolic dysfunction (LVDD) according to the age

In our study of 158 patients, we found that among 116 patients belonging to the age group 31-60 years, 52 had CCM. Of these, 30 patients belonged to the 46-60 years age group. This showed that CCM most commonly occurred in the adult population (Figure 1). Table 1 shows the distribution of baseline characteristics of the variables included in this study. We obtained a significant correlation of LVDD with age (p-value=0.0439).

**Table 1:** Baseline characteristics of the variables included in this study LVDD: left ventricular diastolic dysfunction; HBV: Hepatitis B virus; HCV: Hepatitis C virus

Variables	Range	LVDD absent n (%)	LVDD present n (%)	Total n (100%)
Age group (years)				
	15-30	17 (17.89)	3 (4.76)	20 (12.65)
	31-45	35 (36.84)	22 (34.92)	57 (36.09)
	46-60	29 (30.52)	30 (47.61)	59 (37.34)
	>60	13 (13.68)	9 (14.28)	22 (13.92)
Gender	Male	82 (51.8)	60 (37.97)	142 (89.87)
	Female	13 (8.24)	3 (1.89)	16 (10.13)
Alcohol consumption	Yes	12 (7.59)	5 (3.16)	17 (10.75)
	No	83 (52.53)	58 (36.7)	141 (89.25)
Viral cause	Absent	92 (58.22)	58 (36.7)	150 (94.93)
	HBV	2 (1.2)	4 (2.5)	6 (3.79)
	HCV	1 (0.64)	1 (0.64)	2 (1.28)
Other causes	Absent	91 (57.59)	62 (39.24)	153 (96.83)
	Present	4 (2.53)	1 (0.63)	5 (3.16)

Sex distribution

**Figure 2:**
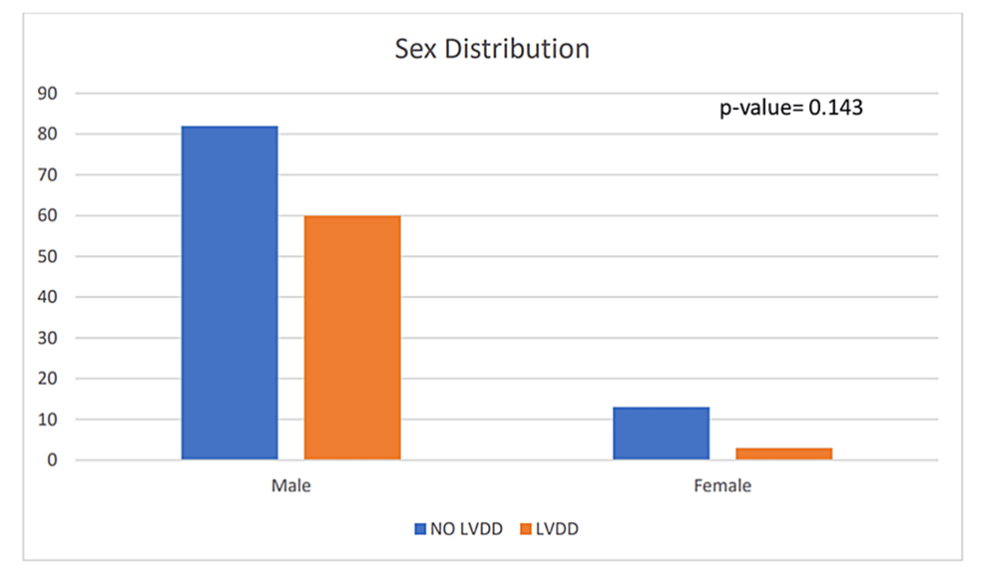
Graph showing the distribution of left ventricular diastolic dysfunction (LVDD) according to the sex

Out of 158 patients, 142 are males and 16 are females. Of 142 males, 60 of them had LVDD, and of 16 females, three had LVDD (Table 1, Figure 2).

A significant correlation between sex and LVDD could not be established from our study (p-value=0.143). Most of the patients who enrolled in the study were males and hence most of the cirrhotic patients with cardiomyopathy were adult males.

Alcohol dependence

**Figure 3:**
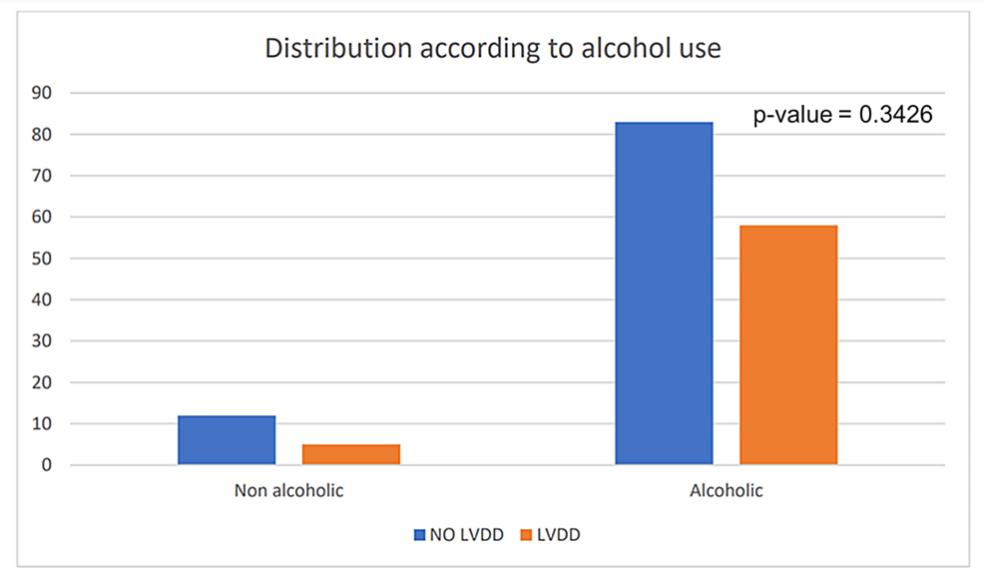
Graph showing the distribution of left ventricular diastolic dysfunction (LVDD) according to the alcohol consumption

In the current cohort, 58 non-alcoholic and five alcoholic study subjects suffered from LVDD. A statistically significant correlation for LVDD could not be drawn from the results of our study since 87.36% of non-alcoholic patients had LVDD as against 12.64% (Table 1, Figure 3) with a p-value=0.3426.

Viral association

**Figure 4:**
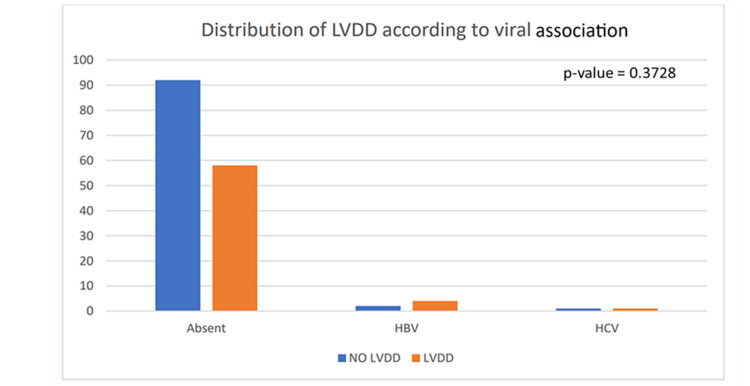
Graph showing the distribution of left ventricular diastolic dysfunction (LVDD) according to the viral cause HBV: Hepatitis B virus; HCV: Hepatitis C virus

We also studied the effect of the Hepatitis B virus (HBV), Hepatitis C virus (HCV), and the viral associations of liver cirrhosis. In this study, four patients infected with HBV and one patient infected with HCV who had cirrhosis developed CCM and the effect of the viral association was not statistically significant for the development of CCM (p-value=0.3728) (Table 1, Figure 4).

Other causes

Among the associated reasons for chronic liver diseases other than alcohol consumption, autoimmune and cryptogenic causes may be responsible, including LV hypertrophy, non-alcoholic steatohepatitis, and metabolic disorders (Table 1). The p-value of 0.3579 reflected that the other causes are not statistically significant for LVDD.

Distribution pattern of hepatic encephalopathy

Out of 158 patients, 105 do not have hepatic encephalopathy and 53 of them have hepatic encephalopathy. Hepatic encephalopathy did not bear a significant correlation with LVDD (p value=0.5219) in our study.

Distribution patterns of biochemical parameters

Serum Bilirubin

**Figure 5:**
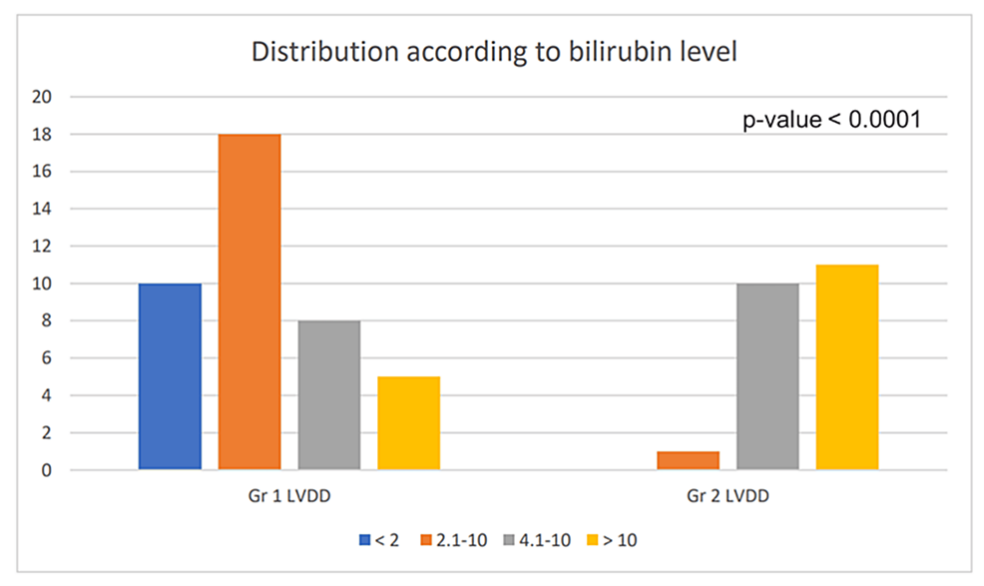
Distribution of left ventricular diastolic dysfunction (LVDD) according to the serum bilirubin level Gr: grade

The majority of patients under our investigation have higher serum bilirubin (>2 mg/dL). Nineteen patients with LVDD have serum bilirubin levels between 2.1 and 4 mg/dL, 18 LVDD patients have levels of serum bilirubin between 4 and 10 mg/dL and 16 have levels more than 10 mg/dL (Figure 5). A total of 41 patients have Grade 1 LVDD and 22 patients have Grade II LVDD. Bilirubin levels bear a significant correlation with LVDD (p-value<0.0001) (Table 2). Here, 13 out of 41 patients with raised bilirubin had Grade I LVDD, and 21 out of 22 patients with high bilirubin had Grade II LVDD and showed a significant correlation with the severity of cardiomyopathy.

**Table 2:** Distribution of left ventricular diastolic dysfunction (LVDD) according to serum bilirubin levels *p-value<0.0001

Echocardiography		Bilirubin (mg/dL)				Total
		<2	2.1-4	4.1-10	>10	
Grade 1 LVDD	No.	10	18	8	5	41
	%	24.39	43.90	19.51	12.19	100
Grade 2 LVDD	No.	0	1	10	11	22
	%	0	4.54	45.54	50	100
Total	No.	10	19	18	16	63
	%	15.87	30.15	28.57	25.39	100

Serum Albumin

**Figure 6:**
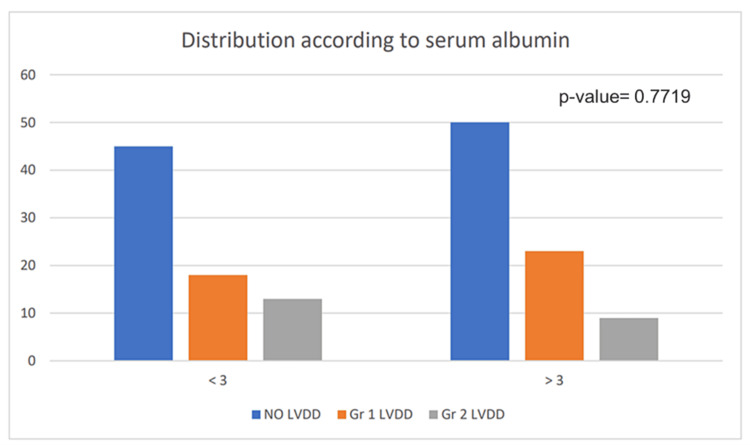
Distribution of left ventricular diastolic dysfunction (LVDD) according to serum albumin levels Gr: grade

Serum albumin is the major blood plasma protein and the major carrier of free fatty acids in the blood. A low level of serum albumin is an indicator of liver disease. Here we evaluated the conditions of the patients according to serum albumin concentration. Out of 158 patients, 76 patients had levels of serum albumin less than 3 mg/dL and 82 patients had serum albumin levels more than 3 mg/dL. Out of 76 patients having serum albumin less than 3 mg/dL, 45 had no LVDD, 18 patients had Grade I LVDD and 13 patients had Grade II LVDD. Out of 82 patients having serum albumin of more than 3 mg/dL, 50 of them had no LVDD, 23 had Grade I LVDD, and nine had Grade II LVDD (Figure 6). Serum albumin levels do not bear a significant correlation with LVDD (p-value=0.7719). In our study, among 158 patients we could not find statistical significance between serum albumin level and LVDD.

Distribution of LVDD according to CTP score

To evaluate LVDD in DCLD, we attempted to find a correlation between CTP score and the grades of LVDD, and hence establish the LVDD as an early indicator of CCM.

**Figure 7:**
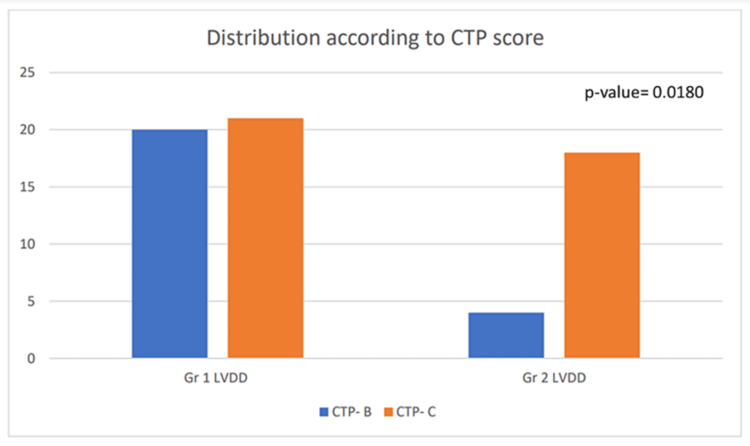
Correlation of left ventricular diastolic dysfunction (LVDD) according to clinical patterns and its distribution according to the CTP grade CTP: Child-Turcotte-Pugh; Gr: grade

**Figure 8:**
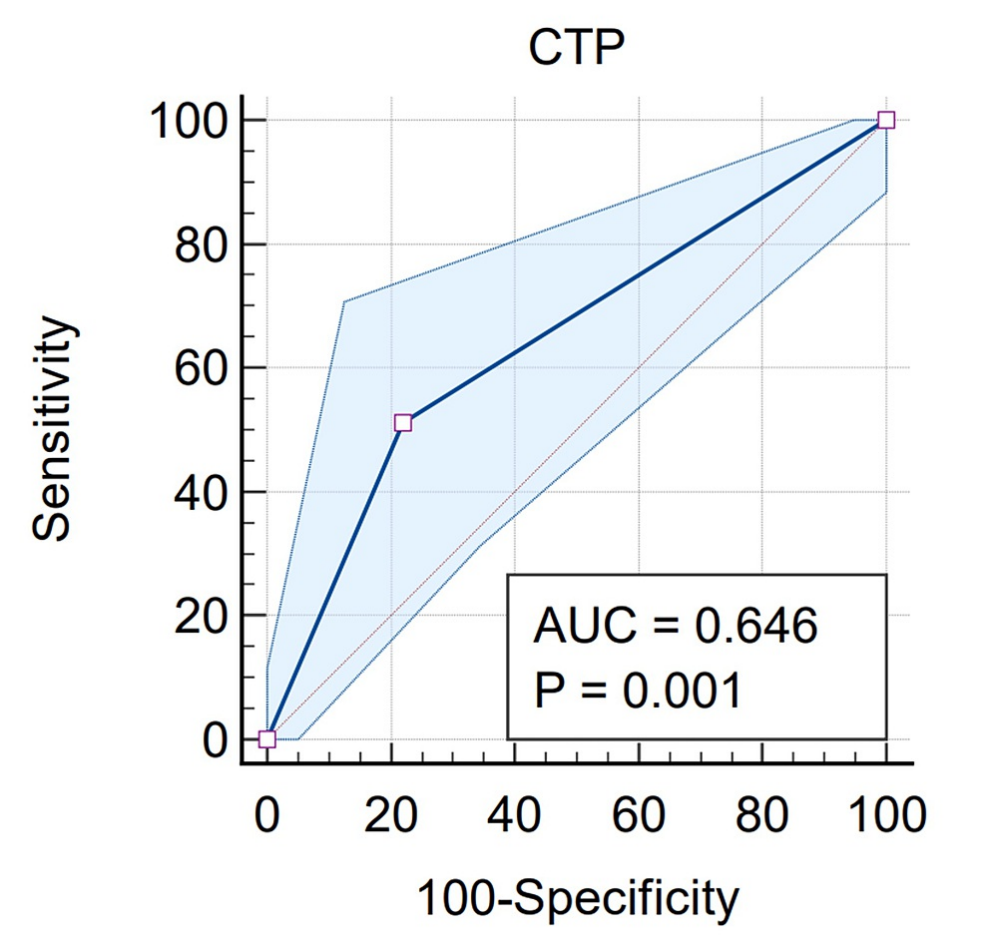
Correlation of left ventricular diastolic dysfunction (LVDD) according to clinical patterns and receiver operating characteristic (ROC) curve showing the correlation between Child-Turcotte-Pugh (CTP) grade and LVDD AUC: area under the curve

Out of the total number of patients having LVDD, 24 of them have Grade I LVDD and 22 of them have Grade II LVDD. Twenty-four patients belong to CTP class B and 39 patients belong to CTP Grade C (Figures 7, 8). CTP class shows a significant correlation with the development of LVDD (p-value=0.0180) (Table 3).

**Table 3:** Distribution of left ventricular diastolic dysfunction (LVDD) according to the Child-Turcotte-Pugh grade *p-value=0.0180

Echocardiography		Child-Pugh scoring system		Total
		B	C	
Grade 1 LVDD	No.	20	21	41
	%	48.78	51.21	100
Grade 2 LVDD	No.	4	18	22
	%	18.18	81.81	100
Total	No.	24	39	63
	%	38.09	61.90	100

Also, upon applying multiple linear regression, we obtained a significant correlation between CTP score and LVDD. The ROC curve shows an AUC of 0.646 (Figures 7, 8) and hence the patient with a higher CTP score has more chances of LVDD.

Distribution of LVDD with QTc interval

All 158 patients were evaluated for QTc prolongation by ECG and LVDD by conventional echocardiography for CCM screening. From the above data, it is inferred that the relationship between the QTc interval and LVDD is statistically significant with a p-value of 0.0001 (Table 4).

**Table 4:** Data showing the presence of QTc interval in left ventricular diastolic dysfunction (LVDD) patients for Grade 1 and Grade 2 conditions *p-value<0.0001

QTc interval	Grade 1 LVDD	Grade 2 LVDD	Total
<400 msec	121	9	130
>400msec	15	13	28
Total	136	22	158

**Figure 9:**
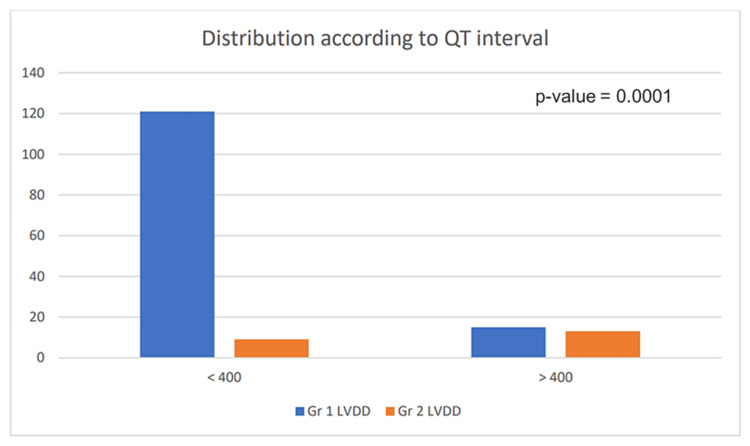
Correlation of left ventricular diastolic dysfunction (LVDD) according to clinical patterns and distribution according to QTc grade Gr: grade

Out of 158 patients, 130 had a QTc interval <400 msec and 28 had a QTc interval >400 msec (Figure 9). A total of 136 patients had Grade I LVDD and 22 patients had Grade II LVDD. QTc interval bears a significant correlation with the development of LVDD.

**Figure 10:**
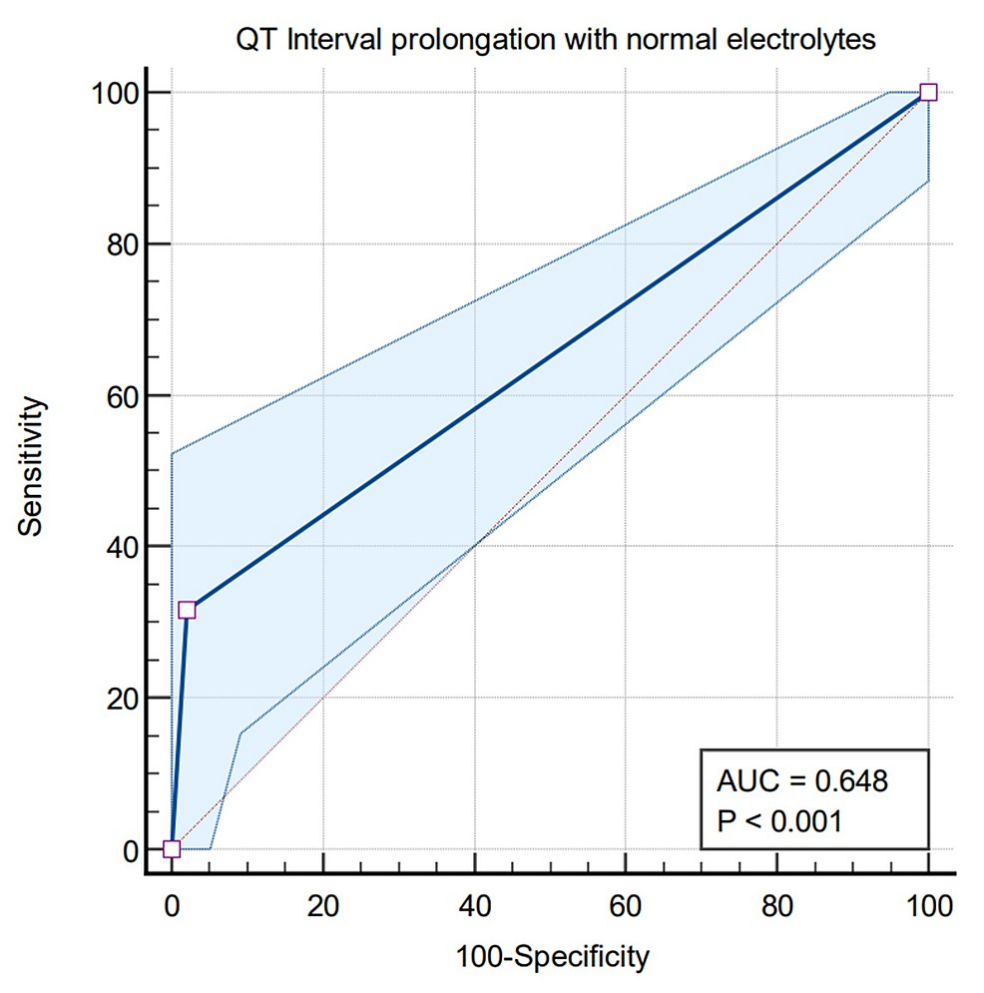
Correlation of left ventricular diastolic dysfunction (LVDD) according to clinical patterns and receiver operating characteristic (ROC) curve showing the correlation between QTc grade and LVDD AUC: area under the curve

The ROC curve obtained upon application of multiple linear regression showed an AUC value of 0.648 (Figure 10). QTc interval prolongation has a significant correlation with the development of LVDD.

## Discussion

Cirrhosis is accompanied by numerous cardiovascular abnormalities. This study was done in patients with DCLD to assess the prevalence of CCM in them and to establish LVDD as an indicator of CCM. The study also aimed to find a relationship between demographical, etiological, biochemical, and clinical parameters with DD in patients of DCLD and understand their pattern of distribution in CCM.

Among 63 patients with CCM, 30 patients (47.61%) belonged to the age group of 46-60 years, reflecting that CCM most commonly occurred in the adult population of this age group. We found that age is associated with a higher prevalence of CCM (p-value=0.0439). Findings of various studies [8-10] have also demonstrated that age is one of the risk factors in such patients though another study by Uyanikoglu et al. [11] has shown no significant correlation between age and CCM.

Of 63 patients with CCM, 60 cases (37.97%) occurred in male patients and three occurred in female patients. Since most of the patients enrolled in our study were males, hence most of the cirrhotic patients with cardiomyopathy were adult males. Most of the studies conducted do not show any significant correlation between sex and LVDD though Belay et al. [8] found female gender to be one of the factors for the higher prevalence.

The cardiovascular changes in cirrhosis of the liver gained importance in the early 1990s when systemic hemodynamic changes were recognized in cirrhotic patients. In the 1950s, hyperdynamic circulation like low arterial blood pressure, low peripheral resistance, and higher cardiac output was observed in alcohol-dependent liver disease patients and hence was attributed to the effect of alcohol [8]. Among the causes of chronic liver diseases, chronic alcohol dependence is a major cause, followed by viruses and a minor fraction including autoimmune and cryptogenic causes. Our study does not show a significant correlation between alcohol consumption and cardiomyopathy (p-value=0.34) among the patients enrolled. Hence, LVDD may not be heavily confounded by alcohol use in our cohort. In this study, four patients infected with HBV and one patient infected with HCV who had cirrhosis developed CCM. Although HCV infection is the most prominent cause of cirrhosis-related global deaths followed by alcohol-associated causes, the burden from the latter is gradually rising, probably due to lifestyle transformations. The advancing HBV vaccination coverage and increased availability of effective anti-HBV antivirals have contributed significantly to a reduction in global death rates [12]. Most patients with HCV infection develop chronic myocardial inflammation, which later manifests itself in dilated cardiomyopathy, necrosis, and finally, loss of myocytes. Velocity of local pulse wave rises in HCV-related liver cirrhosis and correlates with poorer survival in decompensated patients [13]. In our study, the prevalence of alcohol consumption, HBV and HCV association was 3.16%, 2.5% and 0.63% respectively. Our study attributes neither alcohol consumption nor viral association in the development of LVDD here. The role of neither alcohol consumption nor hepatic encephalopathy could be established as a pre-emptive factor for the LVDD here.

Since the study population was dominated by males against females, we were unable to establish the effect of sex in the evaluation. Some studies have indicated CCM to be more common in adult males, over 50-years-old, and in patients with cirrhosis due to alcohol abuse [14]. Nazar et al. attributed the aetiology of cirrhosis to alcohol in 45% of the patients and HCV in 40% [15]. Other causes like autoimmune, cryptogenic, and metabolic disorders could not be correlated (p-value=0.35) with CCM in our study.

Serum albumin mitigates hepatic encephalopathy. Serum albumin levels prominently decrease with the progression of liver disease [16] although in our case of 158 patients, we could not find statistical significance between serum albumin level and LVDD (p-value=0.7719) indicating no relationship with the progression of liver disease.

Grade I LVDD refers to the early relaxation abnormality while Grade II indicates increased atrial filling pressure. We found a significant correlation between serum bilirubin levels and LVDD, with a p-value of 0.0001. A total of 39.8% of the patients had high serum bilirubin levels above 2.1 to more than 10 mg/dL. Nearly, 49.2% and 34.9% of patients with serum bilirubin levels >2.1 mg/dL had Grade I and Grade II LVDD respectively with a total of 84.1%. Hence bilirubin level is an indicator of LVDD.

The severity of hepatic disorder affects the consequence rather than the cardiac dysfunction [17-19]. The association of cardiac dysfunction with the severity of hepatic illness can be biochemically evinced by raised serum bilirubin levels. The progression of liver disease is accompanied by an increase in serum bilirubin and creatinine from grades Child A to Child C. The results from the study by Khurana et al. [20] revealed that 13 out of 41 patients with high bilirubin had Grade I LVDD, and 21 out of 22 patients with raised bilirubin had Grade II DD alongside a significant correlation with the severity of cardiomyopathy. Our investigation has revealed a significant correlation between high serum bilirubin and LVDD. The relationship between echocardiographic pattern and serum bilirubin in the identification of CCM was found to be statistically significant in our study. Our study findings show portal hypertension in 146 patients and hepatic encephalopathy in 23 patients with CCM indicating the possibility of hepatic encephalopathy to be associated with the severity of chronic decompensated liver disease though Scarpati et al. [21] have shown no direct association between hepatic encephalopathy and CCM. Kapoor et al. [22] stated that complications of cirrhosis like hepatic encephalopathy are more common in the CCM group.

CTP’s scoring system is an important predictor of the severity of liver disease. The scores of Class A, Class B, and Class C: 5-6, 7-9, 10-15 indicate least severe, moderately severe, and most severe liver disease respectively. The results from this study showed that 63 (39.8%) out of 158 patients had CCM, 24 patients (38%) belonged to the CTP Class B, and 39 (62%) to the CTP Class C. The distribution of LVDD according to CTP grade displayed that 41 (25.9%) patients had Grade I LVDD and 22 (13.9%) suffered from Grade II. Twenty Child-Pugh B patients had Grade I LVDD and four patients had Grade II DD. Twenty-one Child-Pugh C patients had Grade I LVDD while 18 patients suffered Grade II DD. The significant correlation of LVDD with CTP grades (p-value=0.0180) and AUC value of 0.646 in the multiple regression ROC curve from our investigation points to the severity of LVDD in consonance with liver cirrhosis. ROC analysis is a systematic tool for the quantification of the impact of variability among the individuals' decision thresholds. Studies by Sankar et al. have also demonstrated similar statistically significant results that most of the CCM patients had DD which exists either alone or in combination with other features such as systolic dysfunction and QTc prolongation [23]. Higher Class B and C Child-Pugh scores are indicative of pronounced cardiovascular aberration in cirrhosis [24].

More often, the warning bell of CCM is ‘rung’ by QTc prolongation in electrocardiogram. A significant increase of QTc within normal limits has been found in compensated cirrhotic patients in comparison to the control group [11]. There is a strong correlation between QTc and high serum bilirubin levels and cardiac arrhythmia [17]. Patients with high serum bilirubin have significant QTc prolongation requiring admission to special care units [18]. Echocardiographic parameters viz. QTc intervals are comparable only in CTP B and CTP C grade patients though no significant correlation could be found either between echocardiographic parameters or the QTc interval and hepatic illness. In the current investigation, out of 158 patients, 130 have a QTc interval <400 msec and 28 have a QTc interval >400 msec. A total of 136 patients have Grade I LVDD and 22 patients have Grade II LVDD. We found that the QTc interval bears a significant correlation with the development of LVDD, both as a p-value <0.0001 and an AUC of 0.648 on applying multiple linear regression for the ROC curve. Here, the calculated average QTc interval was 0.63 msec, Class B Child-Pugh had an average QT of 0.57 msec and Class C Child-Pugh had 0.66 msec. Hence both LVDD and QTc interval prolongation are related to severe liver cirrhosis and are also major criteria of CCM. Patients with Child-Pugh C have prolonged QTc interval with most of them showing Grade II DD. LVDD is caused by a delay in the left ventricular filling either due to ventricular hypertrophy, disruption of collagen structure, or subendocardial oedema [11]. Patients with prolonged QTc interval have usually lower survival than their normal counterparts. Corrected QTc interval prolongation is linked not only with severe cirrhosis but also complications of the liver like hepatic encephalopathy and hepatorenal syndrome. The Child-Pugh score bears a statistical relationship with QTc duration [25]. So we advocate that CCM is a grave complication of cirrhosis, whose severity correlates with the stages of liver fibrosis graded by the Child-Pugh system.

Our study is limited by the dominance of males over females in the study population, due to which we could not establish the effect of gender in the evaluation. Grading LVDD with an E/A ratio by conventional echocardiography has certain limitations since it strongly depends on preload and calls for age correction. Hence, pulsed tissue Doppler imaging is the most appropriate modality for assessing LVDD. It is independent of the volume status and also left atrial pressure. Our study method could not subject the patients to further physiological/ pharmacological/surgical/stress challenges. Due to the lack of clinical follow-up, whether CCM affects mortality could not be assessed in the subjects.

With this study, we propose that liver disease severity can be established with the CTP scoring system. Our study reflects that the criteria of the age of the patient above 46 years, serum bilirubin levels >2.1 mg/dL, CTP grades B and C, and abnormal QTc intervals are supportive indicators in the early diagnosis of CCM.

## Conclusions

CCM is a clinically and pathophysiologically distinct condition that is prevalent in 40% of cirrhosis patients who have a normal systolic function at rest. The severity of LVDD has a significant correlation with increasing serum bilirubin levels. The growing severity of liver cirrhosis from the grades of Child-Pugh Classes A to C strongly correlates with the increasing severity of LVDD. The parameters of QTc prolongation and LVDD may aid the diagnosis of CCM in liver cirrhosis patients. The LVDD may serve as a suspect for the onset and progression of liver disease in DCLD. Thus, patients with liver cirrhosis should undergo electrocardiographic and echocardiographic assessment at regular intervals for early detection of LVDD. This could be beneficial in patients at risk to improve overall survival rates.

## References

[1] Kalluru R, Gadde S, Chikatimalla R, Dasaradhan T, Koneti J, Cherukuri SP (2022). Cirrhotic cardiomyopathy: the interplay between liver and heart. Cureus.

[2] Huang DQ, Terrault NA, Tacke F, Gluud LL, Arrese M, Bugianesi E, Loomba R (2023). Global epidemiology of cirrhosis - aetiology, trends and predictions. Nat Rev Gastroenterol Hepatol.

[3] Maron BJ, Towbin JA, Thiene G (2006). Contemporary definitions and classification of the cardiomyopathies: an American Heart Association Scientific Statement from the Council on Clinical Cardiology, Heart Failure and Transplantation Committee; Quality of Care and Outcomes Research and Functional Genomics and Translational Biology Interdisciplinary Working Groups; and Council on Epidemiology and Prevention. Circulation.

[4] Liu H, Naser JA, Lin G, Lee SS (2023). Cardiomyopathy in cirrhosis: from pathophysiology to clinical care. JHEP Rep.

[5] Yoon KT, Liu H, Lee SS (2020). Cirrhotic cardiomyopathy. Curr Gastroenterol Rep.

[6] Glenn TK, Honar H, Liu H, ter Keurs HE, Lee SS (2011). Role of cardiac myofilament proteins titin and collagen in the pathogenesis of diastolic dysfunction in cirrhotic rats. J Hepatol.

[7] Feldman M, Friedman LS, Brandt LJ (2020). Sleisenger and Fordtran's Gastrointestinal and Liver Disease-2 Volume Set, 11th Edition. https://www.us.elsevierhealth.com/sleisenger-and-fordtrans-gastrointestinal-and-liver-disease-2-volume-set-9780323609623.html.

[8] Belay T, Gress T, Sayyed R (2013). Cirrhotic cardiomyopathy among patients with liver cirrhosis. Open J Gastroenterol.

[9] Ginès P, Krag A, Abraldes JG, Solà E, Fabrellas N, Kamath PS (2021). Liver cirrhosis. Lancet.

[10] Lu X, Wang Z, Yang L, Yang C, Song M (2021). Risk factors of atrial arrhythmia in patients with liver cirrhosis: a retrospective study. Front Cardiovasc Med.

[11] Sezgin B, Cindoglu C, Uyanikoglu A, Yenice N (2019). Association of cirrhosis and cardiomyopathy. Euroasian J Hepatogastroenterol.

[12] Huang CH, Wu LS, Jeng WJ, Cheng YF, Ko YS, Sheen IS, Lin CY (2019). In HCV-related liver cirrhosis, local pulse wave velocity increases and in decompensated patients correlates with poorer survival. PLoS One.

[13] Kuna L, Jakab J, Smolic R, Wu GY, Smolic M (2019). HCV extrahepatic manifestations. J Clin Transl Hepatol.

[14] Lyssy LA, Soos MP (2023). Cirrhotic cardiomyopathy. StatPearls [Internet].

[15] Nazar A, Guevara M, Sitges M (2013). LEFT ventricular function assessed by echocardiography in cirrhosis: relationship to systemic hemodynamics and renal dysfunction. J Hepatol.

[16] Is B, Bombassaro IZ, Tovo CV, de Mattos ÂZ, Ahlert M, Chiesa T, de Mattos AA (2021). Albumin in the management of hepatic encephalopathy: a systematic review and meta-analysis. Ann Hepatol.

[17] Arya S, Kumar P, Tiwari B, Belwal S, Saxena S, Abbas H (2020). What every intensivist should know about impairment of cardiac function and arrhythmias in liver disease patients: a review. Indian J Crit Care Med.

[18] Reddy SVC, Boddu J (2015). A study of changes in QTc interval in ECG in cirrhosis of liver. J Evol Med Dent Sci.

[19] Al Atroush HH, Mohammed KH, Nasr FM, Al Desouky MI, Rabie MA (2022). Cardiac dysfunction in patients with end-stage liver disease, prevalence, and impact on outcome: a comparative prospective cohort study. Egypt Liver J.

[20] Khurana S, Raufman JP, Pallone TL (2011). Bile acids regulate cardiovascular function. Clin Transl Sci.

[21] Scarpati G, De Robertis E, Esposito C, Piazza O (2018). Hepatic encephalopathy and cirrhotic cardiomyopathy in Intensive Care Unit. Minerva Anestesiol.

[22] Kapoor N, Mehta V, Singh B, Karna R, Kumar S, Kar P (2020). Prevalence of cirrhotic cardiomyopathy and its relationship with serum pro-brain natriuretic peptide, hepatorenal syndrome, spontaneous bacterial peritonitis, and mortality. Indian J Gastroenterol.

[23] Sankar UMK, Chakravarthy SC, Zeno LFJ, Thirumal P (2020). Prevalence of cirrhotic cardiomyopathy and correlation with Child-Pugh score. Int J Res Rev.

[24] Behera MK, Narayan J, Sahu MK (2021). Factors predicting cardiac dysfunction in patients with liver cirrhosis. Middle East J Dig Dis.

[25] Salari A, Shafaghi A, Ofoghi M, Saeidinia A, Mansour-Ghanaei F (2013). Diastolic dysfunction and severity of cirrhosis in nonalcoholic cirrhotic patients. Int J Hepatol.

